# Reciprocal Modulation of Cognitive and Emotional Aspects in Pianistic Performances

**DOI:** 10.1371/journal.pone.0024437

**Published:** 2011-09-09

**Authors:** Marcia K. Kodama Higuchi, José Fornari, Cristina M. Del Ben, Frederico G. Graeff, João Pereira Leite

**Affiliations:** 1 Department of Neurosciences and Behavior, University of Sao Paulo School of Medicine at Ribeirao Preto, Ribeirao Preto, Brazil; 2 Interdisciplinary Nucleus for Sound Communication (NICS), University of Campinas (UNICAMP), Sao Paulo, Brazil; City of Hope National Medical Center and Beckman Research Institute, United States of America

## Abstract

**Background:**

High level piano performance requires complex integration of perceptual, motor, cognitive and emotive skills. Observations in psychology and neuroscience studies have suggested reciprocal inhibitory modulation of the cognition by emotion and emotion by cognition. However, it is still unclear how cognitive states may influence the pianistic performance. The aim of the present study is to verify the influence of cognitive and affective attention in the piano performances.

**Methods and Findings:**

Nine pianists were instructed to play the same piece of music, firstly focusing only on cognitive aspects of musical structure (cognitive performances), and secondly, paying attention solely on affective aspects (affective performances). Audio files from pianistic performances were examined using a computational model that retrieves nine specific musical features (descriptors) – loudness, articulation, brightness, harmonic complexity, event detection, key clarity, mode detection, pulse clarity and repetition. In addition, the number of volunteers' errors in the recording sessions was counted. Comments from pianists about their thoughts during performances were also evaluated. The analyses of audio files throughout musical descriptors indicated that the affective performances have more: agogics, legatos, pianos phrasing, and less perception of event density when compared to the cognitive ones. Error analysis demonstrated that volunteers misplayed more left hand notes in the cognitive performances than in the affective ones. Volunteers also played more wrong notes in affective than in cognitive performances. These results correspond to the volunteers' comments that in the affective performances, the cognitive aspects of piano execution are inhibited, whereas in the cognitive performances, the expressiveness is inhibited.

**Conclusions:**

Therefore, the present results indicate that attention to the emotional aspects of performance enhances expressiveness, but constrains cognitive and motor skills in the piano execution. In contrast, attention to the cognitive aspects may constrain the expressivity and automatism of piano performances.

## Introduction

The piano performance ability is not a monolithic entity that a person either has or does not have [1]. Piano performance is a domain in which artists accomplish a complex integration of expert motor, perceptual, cognitive, and emotive skills [2]. Nevertheless, studies in neuroscience [3], [4], and music cognitive psychology [5], [6], [7] suggest that such integration of distinct abilities may not occur spontaneously, and imply that cognition and emotion may have antagonistic characteristics.

### The importance of emotion for expressiveness

In the musical environment, the idea that the interpreters' emotion during execution is related to expressiveness is widely accepted. According to some commentators, “the true expressiveness comes ‘from heart’ or is ‘instinctive’ [6]”. However, the role of the performer's emotions during execution is not yet thoroughly understood.

Juslin et al (2010) have proposed seven different mechanisms that can evoke emotional responses to music in listeners. One of the mechanisms proposed, named “emotional contagion”, is supposed to explain the importance of the interpreter's emotion in musical expressiveness. During the processing of an emotion-inducing stimulus, the nervous system activates a sequence of reactions, preparing the body for a specific reaction to each circumstance. Reactions derived from emotions influence many activities, such as body posture, facial expression, blushing, gesticulation, voice intonation [8], [9] and, consequently, change the way the musical instrument is played [6]. These reactions would result in variations of agogics, dynamics, timbre, articulation, among other musical aspects of the performance [6]. The listeners would perceive these emotional expressions and mimic them internally, “by means of periphery feedbacks from muscles, or a more direct activation of the relevant emotional representations in the brain, leading to an induction of the same emotion” [10].

The emotional contagion theory is supported by considerable evidence showing that emotional expression can shape the way the instrument is played, influencing musical aspects, such as: timbre, overall loudness, timing, articulation, vibrato, tone attacks, tone decay and pauses, accent, and interpretative inflections [11], [12], [13]. This hypothesis is supported by reported results showing that professional musicians can play the same music with different expressive nuances, and that both musicians or non-musicians can identify the emotions transmitted by these performances [11], [12], [14], [15]. According to Meyer's theory [16], the listener's perception of expressiveness is induced by either specific violations of certain musical features, such as time delay, or by the confirmation of the listener's expectancy about the music continuation. These inflections are applied by the interpreter without conscious awareness, explaining why expressive musicianship is widely considered to be, intuitive and spontaneous. [7].

### Cognitive Skills in Piano Performance

Piano performance may provide a rich domain for the study of both cognitive and motor skills [14], [17]. In the process of playing the piano, musical units are retrieved from memory and then prepared for production and transformed into movements [14]. The level of awareness in the piano execution process can vary. It can be played with high or low level of awareness of different features. In our previous interactions with piano students, we have observed that some of them played with a high level of awareness and motor control. According to their comments, they played decoding explicitly each note of the score, retrieving from the memory the information about the note localization on the keyboard and play planning and controlling the movement of the each finger. These students presented many difficulties such as automatism and expressiveness constraint, incapacity of playing in time and difficulty to listening to what they are playing. This could explain why the monitoring (realization of whether the execution was performed correctly), was done through visual feedback (they reported that they used to see which notes they were playing to know whether they had played the correct ones). On the other hand, Sloboda (2004) observed that a well known piano piece can be executed automatically by the pianist, without attention being focused on the structural and unitary aspects of the music [18]. According to Sloboda (2004), one of the main problems of average performers is that the performance sequence is dissociated from full conscious control. In this case, each tiny interval of music is guided by the previous one, and in the case of any mistake, there is no way to continue the performance [18]. Higher level performance requires control of many features, besides pitches and tempo. Other aspects, such as dynamics, phrasing and articulation are very important. However, trying to control all these aspects may exceed the attentional capacity of the performer, since the conscious control of each of these skills requires attentional allocation from a limited pool of attention resources. Nevertheless, it seems that expert pianists don't have a broader attention focus. Instead, from their experience, they get to automate all these skills, so that these features are consciously processed in a way that it requires little or no attention for their execution [18].

### Modulation of Cognition by Emotion, and Emotion by Cognition

Observations about reciprocal inhibitory modulation of cognition by emotion and emotion by cognition are not new. In musical learning, the reciprocal modulation between cognition and emotion, or in other words, the antagonism between technique and expressiveness is frequently cited [5], [18]. In this context, it has been reported that there are “expressive” students who generally play inattentively, and students with technical skills that are cool and inexpressive during their performances. However, as far as we know, there have been no specific studies concerning the influence of cognition and emotion in the piano execution.

In the neuroscience field, studies with functional Magnetic Resonance Imaging (fMRI) have demonstrated that cognitive activity can automatically reduce the activation of cerebral areas involved with emotion, such as cingulate gyrus, medial and orbital prefontral cortex (PFC), hippocampus, parahippocampal gyrus, insula, the retrosplenial cortex, brainstem and amygdala [3], [19], [20], indicating that such emotional modulation is related to the amount of attention guided by the cognitive task [20], [21]. Hence, it may be supposed that the piano execution with attention focused on cognitive aspects constrains the performer's emotional state. If we consider the hypothesis that emotion plays an important role in music expressiveness, performances with attention directed to cognitive aspects will probably constrain expressiveness.

On the other hand, in psychology there is a condition named “emotional hijacking”, which occurs when an emotional state overpowers cognition. The physiology of emotional hijacking is partially understood. Amygdala nuclei receive inputs through both a thalamic route, which is independent of attention, and a cortical route, that is affected by attention [3]. Inputs traveling through the thalamic-amygdalar (sub-cortical) route are faster than cortical inputs [22]. Under strong emotion, amygdala output connections alter the functioning of several brain regions that organize adaptive behavioral responses and in this process, cognitive functions may be impaired [23]. Accordingly, reported fMRI results have shown that certain emotional states hamper the performance of cognitive tasks [3], [24]. In these studies, the presentation of pictures related to negative emotional states disrupted cognitive task performance, with increased reaction time in volunteers exposed to emotional as compared to the neutral pictures.

### Objective

Assuming that emotion enhances expressiveness, but hinders cognition, we may expect that affective performance will be more expressive, but will present more errors than performances with attention directed to cognitive aspects. On the other hand, performances with the attentional focus directed to cognitive aspects will make the performer more aware of his/her performance, at the expense of losing expressiveness.

To study the influence of attention to either cognitive or emotional features of performance on piano performance, the present study aims to compare pianists with high expressive and technical skills, executing the same piano piece, firstly with attentional focus on the cognitive aspects (cognitive performance), and then with attentional focus on emotional aspects of performance.

## Materials and Methods

### Participants

We compared four executions of the same musical piece, performed by nine pianists aged from 20 to 36 years (means 25.11±5.25 std. dev.). The pianists are graduate or undergraduate students in music from two institutions: Art Institute of São Paulo State University (UNESP) and Alcântara Machado Art Faculty (FAAM). This study was approved by the Ethics Committee of our institution (Process Number: 12191/2007) and a consent form was obtained from all pianists.

### Repertoire

**Figure 1:**
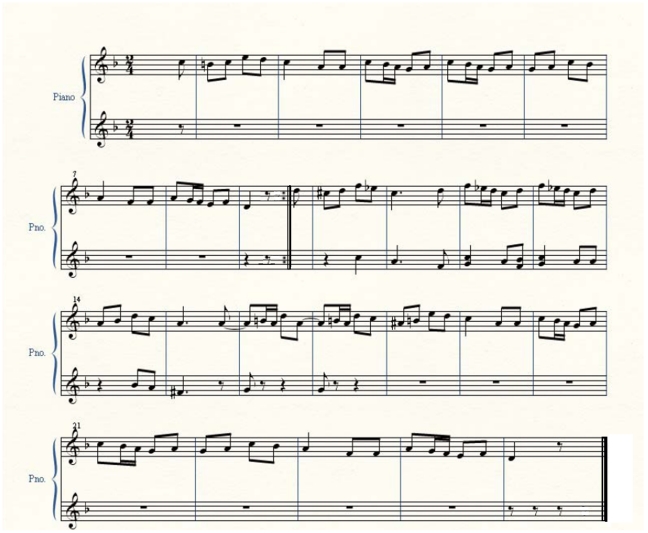
Trauer's primo part score. Primo part that was executed by the volunteers. All the tempo, dynamics and expressive indications were removed from the score in order to avoid induction of any expressive interpretation.

In our pilot study we observed that some piano students presented many difficulties when they played focusing all their attention on planning and monitoring consciously each note they play. In this condition, even graduate piano students made many mistakes when they played a very easy piece, with few notes in the left hand. Therefore, we understood that a difficult repertoire would not be feasible for this experiment. On the other hand, a repertoire with a simple melody, accompanied by few notes in left-hand, would be difficult to be emotionally involved by the music. Therefore we selected for this experiment an adaptation of the first 32 bars of *Trauer* (means sadness in German), in F Major, from the “12 pieces for four-hand piano, for big and little children, Opus 85”, composed by Robert Schumann. The volunteers played the part *primo* (the score is presented in Figure 1) being accompanied by M.K.K. Higuchi (the first author of the present study), who played the part *secondo*. The accompaniment was done in the same conditions, i.e., in the cognitive tasks Higuchi also played focusing her attention on the cognitive aspects of the music, while in the affective tasks, she played aiming to “feel” the music. Higuchi tried not to interfere with the pianists' performances, and played according to the tempo, phrasing and dynamics of the volunteers' interpretations.

### Procedure

Music memorization is not a faculty that can be processed in only one way. Musicians can use different strategies, such as analysis of musical structure, technique, interpretation and expressiveness [25]. As this present study aims to compare the performance with attention focused on different features, it was important that the volunteers memorize the repertoire using different strategies, so that they would be able to play the piece at different levels of awareness.

Hence, the volunteers went through 4 to 5 one-hour long training sessions.

The first session was aimed at the musical piece memorization. The volunteers were asked to consciously learn the music structures and unit, as well to know it implicitly. The following strategies were used in the explicit memorization process: 1) The volunteers were asked to sing naming the notes they played and to sing naming the sequence of notes without playing; 2) They were instructed to repeat the whole piece, dividing it in little parts, playing each hand separately; 3) They were asked to play the part of one hand and sing the part of other hand; 4) The pianists were instructed to repeat the whole piece many times with the eyes closed.

The second session was focused on the musical emotive aspects. Although *Trauer* (the piece title) means sadness in German, this music was not considered to be sad by everyone who listened to it. Therefore, we applied psychological mechanism known as Evaluative conditioning (a process whereby an emotion is evoked by pairing an emotional stimulus with a piece) [10], [26] and a sad emotional stimulus was elaborated to induce the pianists' affect.

The strategies used to improve the volunteer's expressiveness were the following: 1) The volunteers watched the emotional stimulus twice and were asked to play imaging the pictures of the emotional stimulus; 2) They listened to three different expressive interpretation of the same musical repertoire played by João Carlos Martins (a famous Brazilian pianist considered very expressive) with many violations of expectations such as rubatos, ritardandos, subito pianos and unexpected phasing; 3) Meyer's expressiveness theory was explained, and the volunteers were asked to try different interpretations to the same repertoire, violating the interpretation expectancy: 4) They were instructed to elaborate a sad story fit to the music, so that the interpretation could have a sad meaning, and they were asked to play representing the story throughout the music; 6) They were asked to play focusing all their attention to the sadness that the music transmitted.

In the third, fourth and fifth sessions, the volunteers were instructed to play the music in either the cognitive or the affective condition. In the first task, called “cognitive performance”, they were instructed to play thinking about each note they were executing (visualizing the score, planning the movement or thinking beforehand the notes they will play next). As in our pilot study, some volunteers presented difficulties to play thinking about the notes of the both hands, the volunteers of this present study were instructed to think only of the right hand notes in the part where they played with two hands. In the second task, they were instructed to play thinking about the emotional stimulus (imagining the pictures, remembering the associated story or just feeling the music). The volunteers were urged not to play this piece outside of the training sessions.

During the training sessions, the pianists used to make many comments about how they felt, what they were thinking, what happened during the executions, why it happened, and so on.

Each volunteer also had a one-hour long recording session, where they were instructed to play in two distinct manners.

In the first task, called: “cognitive performance”, they were instructed to play focusing their attention on each note they were playing, rescuing explicitly from the memory the sequence of the pitches and rhythms of the musical piece, planning their execution and monitoring each note played.

In the second task, named “affective performance”, they were induced to the emotion of sadness, by the following procedures: 1) Simulating (pretending) the likely physical reactions and melancholic expressions; 2) Remembering personal experiences that had aroused strong feelings of sadness; 3) Watching twice the emotional stimulus. Thereafter, the volunteers were instructed to play the repertory feeling the music and focusing all their attention on the sadness that the musical piece seemed to convey.

After each recorded performance, the pianists were asked whether or not they had succeeded in focusing their attention as previously instructed. All sessions, but two (one training and one recording session) were recorded.

Four recordings of each pianist, two cognitive and two affective, were selected for descriptors analysis using a computational model. The selection criterion for one of the executions of each condition was to choose the recordings the volunteers thought to have better succeeded in focusing the attention on the respective task (these performances were denominated cognitive 1 or affective 1). The selection of a second recording of each condition was done randomly (these performances were denominated cognitive 2 or affective. 2) The performances with errors were not selected, because the errors could influence the performance and consequently might affect the features related to the expressiveness.

### Material

The emotional stimulus consisted of selected photos from the International Affective Picture System (IAPAS) presented with *Trauer* (repertoire of this current study) as background music, played by the pianist João Carlos Martins.

The recording was made using a Steinway & Sons Piano, Series D, with 3 Microphones Neumman KM 184, 2 Microphones DPA 4006, Canaire cables, and a Mackie 32/8 mixer.

### Data Analysis

Three different aspects were analyzed: the volunteers' comments during the training sessions; the descriptors and the volunteers' errors in the recording sessions.

Some of the volunteer's reports during the training sessions were qualitatively described, considering that they were rich in details that could help to clarify many aspects of phenomena related to the influence of cognition and emotion in the pianistic performances.

In order to analyze each pianistic performance, we used eight computational models that were designed to retrieve specific musical features from digital audio files. These models were presented in Fornari J. & Eerola T. [27]. These are algorithms specifically designed to predict variations, during time, for specific an uncorrelated musical cognition features. The output of each music feature model is, therefore, a time series, containing samples of related musical feature, periodically taken, in uniform periods of time. We applied these models to all audio files from the pianistic performances. These are the following eight acoustic descriptors: articulation, brightness, harmonic complexity, event detection, key clarity, mode detection, pulse clarity and repetition. As described in Fornari J. & Eerola T. [27], these are algorithms initially designed as part of MIRToolbox Lartillot [28], developed during the Braintuning project (www.braintuining.fi). Two of these models were later included in MIRToolbox release. They are: Pulse Clarity and Articulation. Both are part of MIRToolbox function “mirpulseclarity”. The purpose of these models is to retrieve a time series describing the continuous measurement of specific musical features, in an attempt of emulating the cognitive ability of the human auditory cortex in perceiving specific musical features along time, such as pulse clarity and articulation.

Pulse Clarity is the descriptor that measures the sensation of pulse in music. Pulse is here seen as a fluctuation of musical periodicity that is perceptible as “beatings”. The measuring scale of this descriptor is continuous, going from zero (no sensation of musical pulse) to one (clear sensation of musical pulse).

Key Clarity is a descriptor that measures the sensation of tonality, or musical tonal center. This is related to the sensation of how much tonal an excerpt of music (a sequence of notes) is perceived by listeners, disregarding its specific tonality, but only focusing on how clear its perception is. KC prediction ranges from zero (atonal) to one (tonal). Harmonic Complexity is a descriptor that measures the sensation of complexity conveyed by musical harmony. In communication theory, musical complexity is related to entropy, which can be seen as the amount of disorder of a system, or how stochastic is a signal. However, here we are interested in measuring the “auditory perception” of entropy, instead of acoustic entropy of a musical sound. The measuring scale of this descriptor is continuous and goes from zero (imperceptible harmonic complexity) to one (clear identification of harmonic complexity).

Articulation usually refers to the way in which a melody is performed, if it is played “detached”(staccato) or “linked” (legato). This descriptor attempts to grasp the articulation from musical audio files and attributing to it an overall grade that ranges continuously from zero (legato) to one (staccato).

Repetition is a descriptor that accounts for the presence of repeating patterns in musical excerpts. These patterns can be: melodic, harmonic or rhythmic. This is done by measuring the similarity of hopped time-frames along the audio file, tracking repeating similarities happening within a perceptibly time delay (around 1 Hz to 10 Hz). Its scale ranges continuously from zero (without noticeable repetition within the musical excerpt) to one (clear presence of repeating musical patterns).

Mode is a descriptor that refers to the major, or Ionian, scale; one of the eight modes of the diatonic musical scale. The most identifiable ones are: major (Ionian) and minor scales (such as the Aeolian). They are distinguished by the presence of a tonal center associated to intervals of major/minor thirds in the harmonic and melodic structure. In the case of our descriptor, MD is a computational model that retrieves from musical audio file an overall output that continuously ranges from zero (minor mode) to one (major mode).

Event Density is the acoustic feature that describes the amount of musical events, simultaneously happening, that are perceived by the listener. There is a cognitive optimum point where listeners can still distinguish distinct musical (rhythmic, melodic or harmonic) events. As an upside down U curve, before and after this point, listeners will perceive less simultaneous events, because of its lack or excess. Its scale ranges continuously from zero (only one identifiable musical event) to one (maximum amount of simultaneous events that an average listener can distinguish).

Brightness is a descriptor that retrieves the synesthetic sensation of musical brightness. It is somewhat intuitive to realize that this aspect is related to the audio spectral centroid, as the presence of higher frequencies accounts for the sensation of a brighter sound. However other aspects can also influence its perception, such as: attack, articulation, or the unbalancing or lacking of partials in the frequency spectrum. Its measurement goes continuously from zero (opaque or “muffled”) to one (bright). A thorough explanation on each descriptor can be found in Fornari & Eerola [29], and Lartillot, Eerola, P.Toiviainen, & Fornari [30].

The intensity of the musical notes was analyzed by the intensity of its waveform (the visual representation of an audio signal, displayed as sound amplitude in time) and by the average amplitude of an audio windows, which was determined by its RMS (Root-mean-square). We calculated the Average RMS and Total RMS Power of audio files amplitude, for all selected audio files of 2 cognitive and 2 affective performances, in order to measure the Loudness of each performance. This measurement – as the ninth acoustic feature of our experiment - was given in attenuation, where 0 dB attenuation means the maximum possible digital amplitude level. The program used to calculate this feature was Adobe Audition 2.0.

By comparing the two audio files of each cognitive and affective task, from the same pianist, it was possible to realize the difference of sound features among performances. The comparison of two performances of each pianist of each condition, selected by different criteria, allow us to know whether the results are related with the specific performance selected or are resulted by the experimental manipulation. The statistical analyses were followed by ANOVA repeated measure with Bonferroni post-hoc test. The p-value that we considered to be statistically significant is p>0.05.

Therefore, the feature differences among two cognitive and two affective tasks were analyzed by nine acoustic features: intensity (loudness), articulation, brightness, harmonic complexity, event detection, key clarity, mode detection, pulse clarity and repetition.

In order to analyze the level of similarity among performances, we calculated the correlation coefficient (R) between 4 performances of each pianist. For each pianist, we calculated the correlation between both cognitive performances, affective performances, and also between cognitive 1 and affective 1 performances; for each music feature retrieved by the loudness, articulation, brightness, harmonic complexity, event detection, key clarity, mode detection, pulse clarity and repetition computer models.

Then we compared, using repeated measures ANOVA, the difference among correlation means between Cognitive 1 and affective 1 (R cognitive 1/R affective 1); cognitive 1 and 2 (R cognitive 1/2); and affective 1 and 2 (R affective 1/2) of all nine pianists for the same features.

Considering the high pianistic skills of all volunteers and the low degree of repertory difficulty, it was observed that two types of errors were more frequent during the training and recording sessions. The volunteers used to play wrong notes and also to forget to play some left hand notes.

We quantified the left hand notes missing error (LH) and wrong notes (pitch error PE) of each volunteer within the recording session and compared them between the cognitive and affective performances. The pitch errors were not measured in semitones. If the pianist played any other note than the right one, independent of the distance between the tones, it would be considered 1 pitch error. As the number of performances of each pianist in each task varies, we divided the amount of errors by the number of executions, for each task. As the variable did not have normal distribution, we applied the non-parametric 2 related sample test (Wilcoxon).

**Figure 2:**
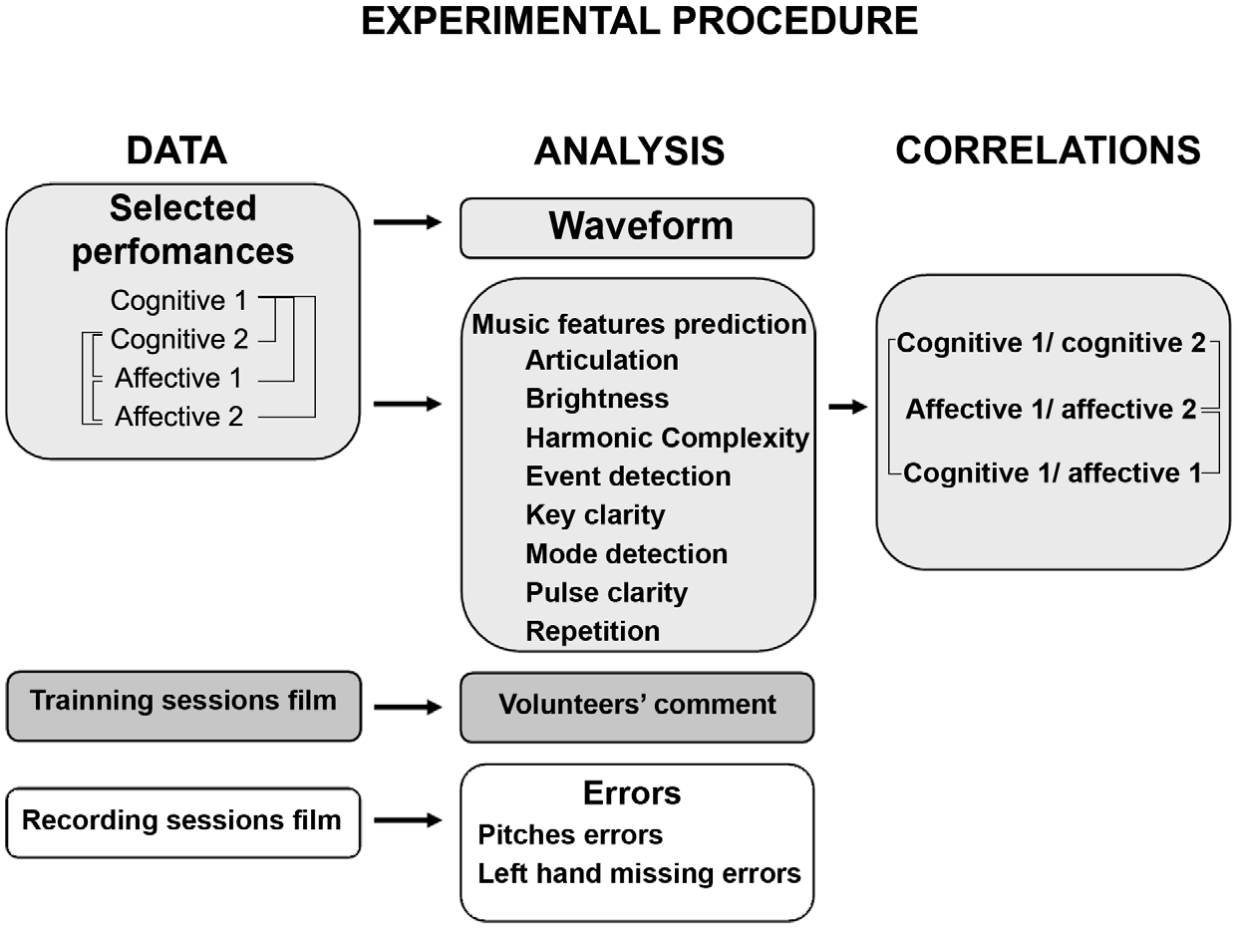
Design of experimental procedure. This diagram schematizes the experimental procedure making a summary of the data, analyzes and correlations used in this study. The lines indicate the comparisons between groups.

Design of experimental procedure is presented in Figure 2.

## Results

### Analysis of Training and Film Recording

The volunteers' descriptions about their perception in the course of training and recording sessions provided relevant information to a better understanding about the influence of cognition and emotion in the musical expressiveness.

Affective execution processing: 8 of 9 volunteers commented that when they played in the affective condition, the cognitive aspects were inhibited. According to their reports, the executions were automatically performed, without their conscious control of movement.

An excerpt from the reports follows below:

“I just remember the first and the last note. The rest goes the way I imagine, so it gets me loose. I just feel sometimes the extremity of the finger, the rest I forget everything. Principally now, I have just played, and I remember the first note […], it seems that it has a fissure in the time and I come back to the reality in the last beat, in D. (volunteer J.P.)”

Another relevant aspect commented by pianists, was the fact that the affective executions differed among them. The alterations in the interpretations were spontaneous and expressed their feelings, reflecting variations in the agogics, phrasing, articulation and timbre of their performances.

Report example:

“If I think it now, I don't know if I am able to do it without instrument, and also I don't know if I play it alone, I would have the same sensation, if I didn't have the accompaniment. That's why I was intrigued. And it seems that it is something that spends much energy when you get in this equilibrium. But it works, it works! The expressiveness increases a lot. I can feel it. It is impressing how the notes (he plays C, B, C, E) they occur in a legato that I can't do without concentrate in this way, without let it go naturally (volunteer F. P.) ”.

Cognitive execution processing: When the volunteers played the piece directing their attention to the explicit and precise execution of each note, the volunteers complained that, in this condition, the expressiveness was very inhibited. They referred to this type of execution as “square”, “mathematical”, “rational”, “pounded”, “mechanical”, and “boring”. Other frequent complains were that this form of processing was mentally very tiresome and the automatism was also inhibited.

Report example:

“How can it interfere so much? In the other one (affective condition) I didn't even see if the other hand entered. No! It entered! I didn't even have to think if it would be the left hand part or not, automatically it would go to play its part. But Now (in the cognitive condition), in the halfway! Where is the left one? How can it happen? It is too much (volunteer A. A.)”.

### Musical Features

The digital audio recording of the selected performances was represented by waveforms and analyzed according to the following acoustic features: intensity, pulse clarity, key clarity, harmonic complexity, articulation, repetition, mode, event density and brightness.

Each one generated a time series, corresponding to the prediction of a specific music feature.

#### Waveform Spectrogram

**Figure 3:**
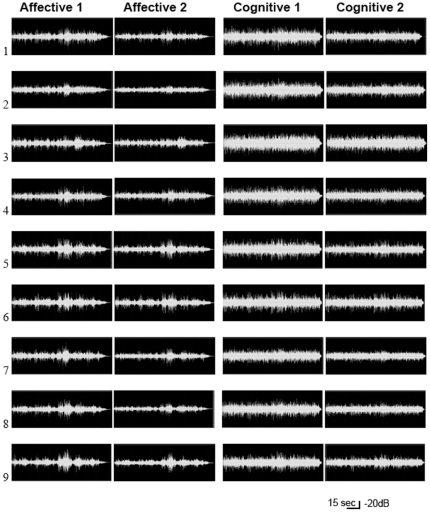
Waveform of the cognitive and affective performances. The recordings show the waveform amplitude of selected cognitive and affective performances of each pianist. As observed, affective performances have greater amplitude variations, when compared to the cognitive ones. The smaller variation of amplitude in the cognitive waveforms indicates less variation of touch intensity in the piano keyboard. This suggests less use of phrasing, which is a fundamental aspect of musical expressiveness.

The waveform amplitude (shown in figure 3) demonstrated that there were more variations of amplitude in the affective performances 1 and 2, when compared to the cognitive ones. More amplitude variation indicates more variations in the dynamics of notes, suggesting more “phrasing”. The comparison of waveforms between performances of the same condition played by the same pianist indicates more amplitude differences in affective performances than in the cognitive ones. Phrasing is related to the expressiveness of a performance: therefore the presence of more phrasing suggests that the affective performances are more expressive than the cognitive ones.

#### Acoustic features

The repeated measures ANOVA of nine acoustic features of the selected performances showed significant differences between cognitive and affective performances in intensity, articulation, event detection and pulse clarity as demonstrated below (respectively in Table 1, 2, 3, 4 and 5). We did not find significant differences among selected performances in key clarity, harmonic complexity, repetition, mode, and brightness. The absence of differences in these 5 features is expected, as key clarity, harmonic complexity, repetition, mode, and brightness are features more related to the composition than to expressiveness.

**Table 1:** 

	Mean	Std. Error	N
Average RMS Power cognitive 1	−21.44	0.30	9
Average RMS Power cognitive 2	−23.54	0.43	9
Average RMS Power affective 1	−26.18	0.17	9
Average RMS Power affective 2	−28.16	0.41	9

**Table 2:** 

	Mean	Std. Error	N
Total RMS Power cognitive 1	−20.86	0.29	9
Total RMS Power cognitive 2	−23.18	0.39	9
Total RMS Power affective 1	−24.90	0.21	9
Total RMS Power affective 2	−26.87	0.47	9

**Table 3:** Descriptive Statistics of articulation of cognitive and affective performances.

	Mean	Std. Error	N
Articulation cognitive 1	1938.65	38.15	9
Articulation cognitive 2	1978.06	30.48	9
Articulation affective 1	1305.83	34.01	9
Articulation affective 2	1338.41	49.20	9

**Table 4:** Descriptive Statistics of event density of cognitive and affective performances.

	Mean	Std.Error	N
Event density cognitive 1	56.00	1.54	9
Event density cognitive 2	53.66	1.23	9
Event density affective 1	50.00	1.25	9
Event density affective 2	48.33	0.50	9

**Table 5:** Descriptive Statistics of Pulse Clarity of cognitive and affective performances.

	Mean	Std. Error	N
Pulse Clarity cognitive 1	39.22	1.17	9
Pulse Clarity cognitive 2	40.00	0.40	9
Pulse Clarity affective 1	32.00	0.66	9
Pulse Clarity affective 2	32.70	0.35	9

#### Intensity

We found significant differences in both the average [F (3; 24) = 125.4 p<0.001] and total RMS power [F (3; 24) = 89.89 p<0.001], which is related to loudness – the perception of waveform amplitude variation. According Bonferroni post-hoc test, all the groups (cognitive 1>cognitive 2>affective 1>affective 2) in both average and total RMS power differed from each other at p<0.01. Both cognitive performances had higher overall loudness, when compared to the affective ones, suggesting that pianistic touches were more intense in the cognitive performances than in the affective ones. The results are shown in the Tables 1 and 2.

Table 1 and 2: Mean and standard error of average (Table 1) and total (Table 2) RMS power of cognitive 1, cognitive 2, affective 1 and affective 2 performances indicate higher overall loudness in both cognitive performances (p<0.01), when compared to the affective ones. The numbers are negative, because the Full Scale Square Wave is equal to 0 dB.

#### Articulation

We found significant differences in articulation [F(3;24) = 135 p<0.001] among 4 performances analyzed. According to Bonferroni post-hoc test, there was less articulation (suggesting more *legatos*, less *staccatos*) in both affective performances of each pianist, when compared to the cognitive performances at p<0.001. We did not find significant differences neither between the two affective performances nor between the cognitive ones. The results are show in Table 3.

Table 3: Mean and standard error of articulation of cognitive 1, cognitive 2, affective 1 and affective 2 performances indicate more articulation in both cognitive performances when compared to the both affective ones (p<0.001). Articulation descriptor overall grade ranges continuously from zero (legato) to one (staccato). Therefore the result suggests more legatos in affective performances than in the cognitive ones.

#### Event Density

We found significant differences in event density among the performances analyzed [F(3;24) = 131 p<0.001]. According to Bonferroni post-hoc test, there was less event density (meaning, smaller amount of perceived musical events) in all the affective performances of each pianist, when compared to their respective cognitive performances (cognitive 1 and affective 1, p = 0.007; cognitive 2 and affective 2, p = 0.013). We did not find any significant differences neither between the two affective performances nor between the cognitive ones. The results are shown in the Table 4.

Table 4: Mean and standard error of event density of cognitive 1, cognitive 2, affective 1 and affective 2 performances indicate more event density in cognitive performances when compared to the respective affective ones (cognitive 1/affective 1, p = 0,007; and cognitive 2 and affective 2, p = 0.013). Event Density descriptor overall grade, ranges continuously, in a unipolar normalized scale, from 0 (one single musical event perceived) to one (the maximum amount of distinct musical events, that can be perceived by the auditory cognition). Therefore, the results point to the existence of simpler perceptual musical features in affective performances, than in its correspondent cognitive ones.

#### Pulse Clarity

We found significant differences in pulse clarity among the performances analyzed [F(3;24) = 41.27 p<0,001]. According to Bonferroni post-hoc test, there was less pulse clarity (suggesting less metric precision or musical meter) in both affective performances, when compared to the cognitive performances at p<0.01. We did not find any significant differences neither between the two affective performances nor between the cognitive ones. The results are shown in the Table 5.

Table 5: Mean and standard error of pulse clarity of cognitive 1, cognitive 2, affective 1 and affective 2 performances indicate more metric precision in both cognitive performances when compared to the affective ones (p<0.001). Pulse Clarity descriptor overall grade ranges continuously from zero (no sensation of musical pulse) to one (clear sensation of musical pulse). Therefore the result suggests more pulse clarity (suggesting less metric precision or musical meter) in both affective performances of each pianist, when compared to the both cognitive ones.

In summary, we have found significant differences in three musical features analyzed (articulation, pulse clarity and event density). There was less articulation (suggesting more legatos, less staccatos), less event density (suggesting simpler perceptual musical textures), and less pulse clarity (suggesting less metric precision or musical meter) in all the affective performances of each pianist, when compared to their respective cognitive ones. Reduction of perceived musical events in affective performances can be due to less articulation. As the notes played in legato are connected in time, they are likely to be perceived as a single musical event. Hence, it is possible that more legato will result in less event density.

The legato articulation and time variations are features related to musical expressiveness. More legatos and less metric precision indicate that the affective performances have more expressive features than the cognitive ones. These data also suggest that these two important musical features, related to the expressiveness were suppressed in the piano performance executed with attention focused on the cognitive aspects of music.

### Correlations

Next, we calculated the correlation coefficient (R) between the same pianist's cognitive performances, between the same pianist's affective performances, and also between same pianist's cognitive 1 and affective 1 performances for each musical feature, namely, loudness, articulation, brightness, harmonic complexity, event detection, key clarity, mode detection, pulse clarity and repetition features.

Then we compared the correlation means (R cognitive 1/R affective 1), (R cognitive 1/2) and (R affective 1/2) of the nine pianists for the same features.

The repeated measures ANOVA showed significant differences among correlation means of intensity feature [F(2;16) = 23.91 p<0.001], articulation [F(2;16) = 4.21 p = 0.03] and pulse clarity [F(2;16) = 3.02 p = 0.07].

#### Intensity

The post-hoc Bonferroni test showed significant differences in intensity between (R cognitive 1/2) and (R cognitive 1/affective 1), at p = 0.02, and between (R affective 1/2) and (R cognitive 1/affective 1) at p<0.001. We did not find differences between (R cognitive 1/2) and (R affective 1/2).

The results are shown in the Table 6.

**Table 6:** Descriptive Statistics of Intensity correlations.

	Mean	Std. Error	N
R cognitive1/R cognitive 2	0.58	0.04	9
R affective 1/R affective 2	0.72	0.01	9
R cognitive 1/R affective 1	0.39	0.02	9

Table 6: Mean and standard error of intensity (R affective 1/2), (R cognitive 1/R affective 1) and (R cognitive 1/2). The post-hoc Bonferroni test indicates significant difference between (R affective 1/2) when compared to (R cognitive 1/R affective 1) at p<0.001, but did not indicate significant difference for (R cognitive 1/2). The (R cognitive 1/2) was also significantly different from (R cognitive 1/R affective 1) at p = 0.02. The mean 0.72 indicates strong association between affective 1/2 performances and the means 0.58 and 0.39 indicate weak positive association between cognitive 1/2 and cognitive 1/affective 1 performances of the respective pianists.

#### Articulation

The post-hoc Bonferroni test showed nearly significant differences in articulation between correlation means for (R affective 1/2) and (R cognitive 1/R affective 1) at p = 0.07, but did not indicate significant differences either between (R cognitive 1/2) and (R cognitive 1/R affective 1) or between (R cognitive 1/2) and (R affective 1/2).

The results are shown in Table 7.

**Table 7:** Descriptive Statistics of Articulation correlations mean.

	Mean	Std. Error	N
R cognitive 1/R cognitive 2	0.07	0.06	9
R affective 1/R affective 2	0.20	0.03	9
R cognitive 1/R affective 1	−0.007	0.05	9

Table 7: Mean and standard error of articulation (R affective 1/2), (R cognitive 1/r affective 1) and (R cognitive 1/2). The post-hoc Bonferroni's test indicates low level of correlation in (R affective 1/2) but still higher than the correlation of (R cognitive 1/r affective 1) p = 0.07 There was no significant difference for (R cognitive 1/2).

#### Pulse Clarity

The post-hoc Bonferroni test showed significant differences in pulse clarity between correlation means of (R affective 1/2) and (R cognitive 1/r affective 1) p = 0.04. We did not find differences either between (R cognitive 1/2) and (R cognitive 1/R affective 1) or between (R cognitive 1/2) and (R affective 1/2).

The results are shown in the Table 8.

**Table 8:** Descriptive Statistics of Pulse Clarity correlations mean.

	Mean	Std. Error	N
R cognitive1/R cognitive 2	0.03	0.07	9
R affective 1/R affective 2	0.19	0.03	9
R cognitive 1/R affective 1	0.03	0.03	9

Table 8: Mean and standard error of correlation mean of pulse clarity between (R affective 1/2), (R cognitive 1/r affective 1) and (R cognitive 1/2). The post-hoc Bonferroni's test indicates low level of correlation of pulse clarity in (R affective 1/2) but still higher than correlation of performances cognitive 1/affective 1 (p = 0.04).

A summary statistics with interactions of all Musical feature predictions (presented in Table S1) and their correlations (presented in Table S2) are shown in supporting information file.

### Errors

**Figure 4:**
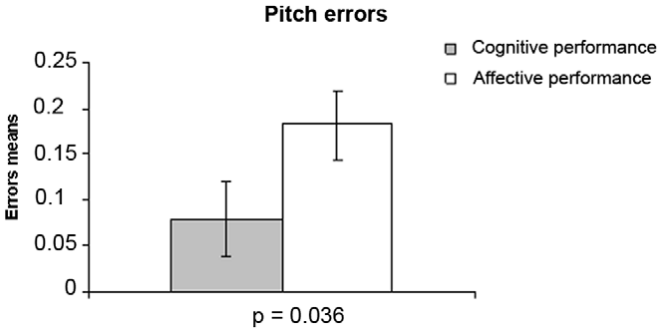
Pitch errors in cognitive and affective performances. Pitch errors mean and standard errors in cognitive (gray column) and affective (white column) performances show more pitch errors in affective than in cognitive performances (p = 0.036).

**Figure 5:**
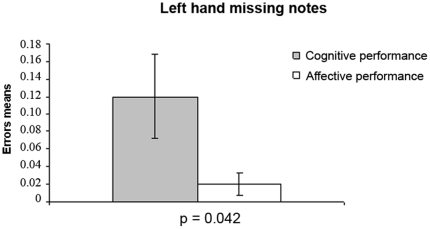
Left hand missing notes in cognitive and affective performances. Left hand missing notes mean and standard errors in cognitive (gray column) and affective (white column) performances demonstrate more left hand missing errors in cognitive than in affective performances (p = 0.042).

We found significant differences in the number of pitch errors (Figure 4) and left-hand missing notes (Figure 5). The results are presented in the graphics below:

The difference in the number of pitch errors between cognitive and affective performances is significant (p = 0.036). As expected, there were more pitch errors (PE) in the affective performances as compared to the cognitive ones suggesting less motor control. According to the volunteers' comments, these errors were caused by the obstruction of their capacity of thinking. Report example: “In that moment that I played to the wrong part, it happened simply because I could not think about what I was playing any more” (volunteer G.S.).

We also found a significant difference (p = 0.042) in the quantity of left-hand missing notes error. There were more errors in the cognitive performances when compared to the affective performances.

Volunteers commented that frequent error in the cognitive performances, such as forgetting to play the notes of the left-hand, was the result of excessive attention to the execution on each note of the right-hand.

Report example: “I thought so much about the right hand that I forgot the left one” (volunteer D. R.).

## Discussion

Although the emotion of the performer is generally considered to be very important in music expressiveness [6], [31], and cognitive aspects were studied in music performance [14], [17], [32], [33], [34], we have not found in the literature any study concerning the reciprocal influence between cognition and emotion in music performance.

In the present work, we have documented both quantitative and qualitative differences between affective and cognitive performances. The main findings of our study suggest that: (1) as the volunteers commented, affective performances seem to inhibit the cognitive aspects of piano execution, while cognitive performances seem to inhibit the expressive aspects of piano execution; (2) affective performances show more agogics, legato and dynamic variation, when compared to the cognitive ones; (3) the volunteers missed more left-hand notes in the cognitive performances than in the affective ones, indicating automatism inhibition; (4) the volunteers played more wrong notes in the affective performances than in the cognitive ones, indicating psychomotor control inhibition.

### The influence of Attention on Emotion in the Piano Performances

The pulse clarity descriptor and the pitch errors analysis indicated that the selected affective performances presented less pulse clarity and motor control when compared to their correspondent cognitive performances. According to the pianists, the affective performances were executed in atemporal, automatic, unconscious manner, without explicit, cognitive and motor control. The affective executions differed from each other, and alterations in the interpretations were spontaneous and supported the expressiveness.

The results obtained with the descriptor-prediction, correlation, waveform and errors analyses are consistent with the above volunteers' comments on affective performances process. In particular, the results with pulse clarity descriptors showing less metric precision and weak correlation between affective performances (indicating little or no association between pulse clarity of affective performances) corroborate the idea of “atemporality” in affective performances. According to cognitive psychology, time perception and timing involve cognition, and attention captured by emotional features may divert processing resources away from the timing system [35]. Therefore, atemporality and greater quantity of pitch errors in the affective task agrees with the idea of impairment of explicit, cognitive and motor control.

The emotional contagion theory assumes that affectivity of the performer is involved in the execution of proper movements to express emotion in pianistic execution. Sad expression is associated with legato articulation, lower sonic amplitudes, large time variation, and flat micro-intonation [11], [12], [15]. The waveform and descriptor analyses suggest that there are more expressiveness features (legatos, agogics, lower sound intensity and musical phrasing) in affective, rather than in cognitive performances, thus corroborating the hypothesis that emotion plays an important role in pianistic expressiveness.

Some expressive students present mind blockade during musical learning, affecting attention, concentration and explicit memory [5]. The experiences reported by the volunteers when they played in the affective condition agree with the characteristics of these “expressive” music students, who present a strong emotional involvement with music and generally play inattentively. This coincidence suggests that inhibition of cognitive aspects and facilitation of expressiveness in musical execution could be a result of experienced emotion. Inhibition of cognitive processes in affective performances could thus be explained by emotional hijacking, once all volunteers reported that they succeeded in directing their attention to the feeling that the music piece transmitted, during their affective performances.

Emotional hijacking may also be involved in motor control inhibition. Experimental studies in rats [36], [37] have shown that injection of anxiogenic drugs in the amygdala can favor caudate-dependent habit learning (automatic) over hippocampus-dependent explicit learning. This data corroborate the pianists'reports that emotion impairs cognitive and motor control features during musical execution, thereby releasing habit at the expense of cognitive memory.

The pianists reported that the affective performances differed from each other and were executed in an atemporal, automatic and unconscious way. Although the correlations demonstrate strong association between affective performances in intensity, the weak correlations in pulse clarity and articulation sustain the existence of differences between affective performances. On the other hand, the comparison of correlations among cognitive and affective performances showed that the association between affective performances is higher than that between cognitive and affective performances in intensity, articulation and pulse clarity. The waveforms also indicate that the variation of intensity keep a pattern suggesting that these differences are not at random. Functional MRI studies [20] suggest that processing of emotional connotation stimulus may reflect the motor activity. Hence we can speculate that the emotions can modulate the motor behavior, corroborating the emotional contagion theory.

Variations in expressive performances have been described in pianistic literature. Many pianists have reported that they “created and recreated” their interpretation when in peak or flow experience [38]. According to contemporary listeners “Frederic Chopin never played his own compositions twice alike, but varied each according to the mood of the moment […] the result was always ideally beautiful” [39].

### The Influence of Attention on Cognition in Piano Performance

Another observation that strengthens the idea that emotion can be important for musical interpretative expressiveness is the fact that when the pianists played the piece directing their attention to cognitive aspects, they complained that their expressiveness was inhibited. The results of descriptor analysis corroborate the idea of expressive inhibition, since it has shown less legatos, more event density and less agogics in the cognitive performances when compared to the affective ones. Less legato implies increase of perceived musical events. As the notes played in staccato are not connected in time, they are not perceived as a single musical event. Hence, more staccato would imply in more event density.

The waveform amplitude variation analysis has demonstrated that cognitive performances have less intensity variation in note striking, and the average and total RMS power analysis has demonstrated that the perceived sound intensity of these notes were stronger in the cognitive performances. This decrease in variation suggests less phrasing, an important feature of musical expressiveness [6], [7], [12], [14], [31], [40]. Less phrasing, less agogics and stronger intensity of note striking result in perception of more event density, matching the volunteers' qualification of cognitive performances as “square”, “mathematical” , “rational”, “pounded”, “mechanical” and “boring”.

Another important information reported by the pianists was that the cognitive performances were mentally tiresome and inhibited automatism. The practice of regulating emotion by cognition is widely known in cognitive psychology [3], [4], [41], [42], [43], [44], [45]. Several regions of the frontal cortex, such as lateral, orbital and dorsolateral areas that perform complex cognitive tasks such as working memory are also involved with emotional regulation, modulating amygdala activity [3], [4], [20], [42], [45]. In the present study, consciously planning and monitoring each note played is a working memory task. The amygdala is a fundamental cerebral structure for the integration between sensorial information and emotional reaction. According to the emotion contagion theory, expressiveness would be the result of emotional reaction. Hence, if cognition regulates emotion and emotion is important for expressiveness, it may be understood why three important features related to musical expressiveness (phrasing, legato and agogics) were suppressed during piano performances executed with attention focused on cognitive musical aspects. Another important information reported by the pianists was that the cognitive performances were mentally tiresome and inhibited automatism.

The analysis of errors showed that the volunteers miss left-hand notes more during cognitive than affective performances. Moreover, according to the same volunteers, this type of error was the result of excessive attention to the execution of each note by the right hand plus automatism inhibition.

We have found in the psychology literature, theories that, overall, can explain why errors have occurred during the cognitive performances. The attention capacity is limited. When the information is rescued from memory in a detailed form, it causes a decrease in the capacity of perception and processing of further information [46].

Cognitive performances request the attention to be focused on each note that is being played. The retrieval of the sequence of notes from memory, and the motor control of each note, directed in a very conscious way to each specific piano key, demands high resolution information. Reasoning tends to occupy a great part of attention [46], little space remaining for the conscious perception of other stimuli. Attention, like the spotlight or the zoom lens, can be either used over a little area with high resolution or distributed over a more extensive area with loss of detail [47]. Therefore, the excessive attention on the execution of each note by the right hand (high resolution over a little area) would use a great deal of attention, leaving to oblivion the execution of the left hand notes.

In experimental psychology studies, the constrained-action hypothesis [48], [49] proposes that when performers make use of an internal focus of attention (focus on the movements) they will constrain or interfere with automatic control processes that would normally regulate movement [48]. Higuchi [50] has observed that piano students that play focusing their attention on inner cognitive aspects have their automatism restrained, corroborating the constrained-action hypothesis. On the other hand, students who played intuitively, spread their attention over extensive areas, thus processing and associating a large amount of information with low resolution, guide their performance by implicit (automatism) and auditory memories (external focus of attention). In the same direction, McNevin et al (2003) [49], have suggested that the distance from an external focus of attention (focus on the movement effect) may allow the performance to be mediated by an automatic control process. The indication that the distance of external attention allows performance to be mediated by automatism may suggest that the attention spread over a more extensive area may be important for the automatism process.

In the present study, although the descriptor results suggest more metric precision in cognitive performances compared to the affective ones, it is interesting to observe that the correlation between cognitive performances in pulse clarity is very weak. Intuitively if there is more metric precision, we would expect more regularity of tempo, but correlation between cognitive performances does not diverge from the correlation between cognitive and affective performances. Therefore, it seems that although the cognitive performances present more pulse precision, this pulse does not keep a pattern. This phenomenon may be due to the inhibition of the automatic control process by the internal focus of attention required by the cognitive condition, which emphasizes adherence to processing at the individual note level. Since high resolution information requires high mental demand and the cognitive condition is a working memory load task, it is easy to understand why the pianists claimed that the cognitive performances were mentally tiresome.

An electroencephalogram (EEG) study revealed differential event-related potential (ERP) response in cognitive vs affective music judgment. This supports the hypothesis that awareness and attention towards different aspects of music may influence performance [51]. Overall, the present results indicate that emotion and cognition influence pianistic execution in distinct ways. Emotion seems to enhance expressiveness and constrain cognitive and motor control. On the other hand, cognition appears to retrain expressivity and constrain automatism. These results corroborate the idea that technique and expressiveness may have an antagonistic interplay in piano execution.

Inherent to our finds, we would like to highlight the limitation of this present work.

Although the analyzes of the volunteers' comments may seem too subjective for a scientific study, we considered these as important data, specially because they provide meaningful information to clarify many aspects of the phenomena related to the influence of attention in the cognitive and affective aspects of pianistic performances. Another reason to analyze these data, concerns the fact that the information we obtained from the volunteers reports are data that could not be achieved by any other way, other than their own comments. We acknowledge that the analysis of the volunteers comments was not well structured. This is due to the fact that all these comments were done spontaneously, as the volunteers were not instructed to report them. As we were not expecting these comments, we have not structured their analysis.

We understand that a piano solo piece would be more adequate for repertory of this present study, because there would be no interference of the researcher. But the utilization of a piano repertory for four-hands was due to the characteristic of the tasks. As we have mentioned before, the cognitive performances require an intense attentional focus to the execution of each note of the right-hand parts. The results of left-hand missing notes errors demonstrate that the use a repertory containing many notes in the left hand would not be feasible. A repertory with a simple melody, accompanied by few notes in left-hand, would be difficult to express the emotion of sadness as we could realize by one of the volunteers comment: “If I think it now, I don't know if I am able to do it without instrument, and also I don't know, if I had played it alone, I would have the same sensation; if I didn't have the accompaniment.”

We acknowledge the fact that the participation of the researcher accompanying the pianists may be a confounding variable, however, the interference of the accompaniment in the results seems unlikely, since the primo part led the execution of tempo, intensity and articulation of the piece.

### Concluding Remarks

The comprehension of the influence of cognition and emotion on activities that require perceptual, motor, cognitive and emotive skills integration may have important implications for several areas of knowledge, such as cognitive neuroscience, psychology and music education.

Cognitive neuroscience [3], [4] has been studying the reciprocal influence of cognition and emotion in simple cognitive tasks, but its influence on activities that require complex integration is not as well understood. The present results indicate that pianistic execution may be an important tool to explore this question.

The present results may be important to understand many of the difficulties found in music learning and performance. A better comprehension of cognitive-emotional integration may guide the search of better procedures for pianistic learning.

## Supporting Information

Table S1 **Summary statistics with interactions of all Musical feature predictions of all variables studied.** We have found significant p-value in average and total RMS power, articulation, event detection and pulse clarity. * We have also found significant p-value in harmonic complexity, but as the difference among the means were not significant (cognitive 1 mean 48.7; cognitive 2 mean 47.4; affective 1 48.2; affective 2 46.4), we considered these differences irrelevant.
(DOC)

Table S2 **Summary statistics with interactions of all correlations statistics of all variables studied.** We have found significant p-value in intensity and pulse clarity. We also found a near significant difference in articulation.
(DOC)

## References

[1] Peretz I, Coltheart M (2003). Modularity of music processing.. Nature Neuroscience.

[2] Parsons LM, Sergent J, Hodges DA, Fox PT (2005). The brain basis of piano performance.. Neuropsychologia.

[3] Blair KS, Smith BW, Mitchell DG, Morton J, Vythilingam M (2007). Modulation of emotion by cognition and cognition by emotion.. Neuroimage.

[4] Northoff G, Heinzel A, Bermpohl F, Niese R, Pfennig A (2004). Reciprocal modulation and attenuation in the prefrontal cortex: an fMRI study on emotional-cognitive interaction.. Hum Brain Mapp.

[5] Gainza VHd (1988).

[6] Sloboda J, Davidson J, Deliège I, Sloboda John (2003). The young performing musician. Musical beginnings: origins and development of musical competence.

[7] Sloboda JA (2005). Exploring the musical mind: cognition, emotion, ability, function.

[8] Ekman P (1973). Darwin and Facial Expression.

[9] Darwin C (2000). A Expressão das Emoções no Homem e nos Animais.

[10] Juslin PN, Vastfjall D (2008). Emotional responses to music: the need to consider underlying mechanisms.. Behav Brain Sci.

[11] Juslin PN (1997). Emotional communication in music performance: A functionalist perspective and some data.. Music Perception.

[12] Juslin PN (2000). Cue utilization in communication of emotion in music performance: Relating performance to perception.. Journal of Experimental Psychology-Human Perception and Performance.

[13] Karlsson J, Juslin PN (2008). Musical expression: an observational study of instrumental teaching.. Psychology of Music.

[14] Palmer C (1997). Music performance.. Annu Rev Psychol.

[15] Juslin PN, Sloboda JohnA (2005). Music and emotion: theory and research.

[16] Meyer LB (1956). Emotion and Meaning in Music.

[17] Palmer C, Drake C (1997). Monitoring and planning capacities in the acquisition of music performance skills.. Can J Exp Psychol.

[18] Sloboda JA (2004). The musical mind: the cognitive psychology of music.

[19] Pallesen KJ, Brattico E, Bailey C, Korvenoja A, Koivisto J (2005). Emotion processing of major, minor, and dissonant chords: a functional magnetic resonance imaging study.. Ann N Y Acad Sci.

[20] Pallesen KJ, Brattico E, Bailey CJ, Korvenoja A, Gjedde A (2009). Cognitive and emotional modulation of brain default operation.. J Cogn Neurosci.

[21] Pessoa L, Padmala S, Morland T (2005). Fate of unattended fearful faces in the amygdala is determined by both attentional resources and cognitive modulation.. Neuroimage.

[22] LeDoux JE (1996). The emotional brain: The mysterious underpinnings of emotional life.

[23] Goleman D (2001).

[24] Pereira M, Oliveira L, Erthal F, Joffily M, Mocaiber I (2010). Emotion affects action: Midcingulate cortex as a pivotal node of interaction between negative emotion and motor signals.. Cognitive, Affective, & Behavioral Neuroscience.

[25] Chaffin R, Lisboa T, Logan T, Begosh KT (2010). Preparing for memorized cello performance: the role of performance cues.. Psychology of Music.

[26] Juslin JNLS, Västfjäll D, Lundqvist LO, Patrik J, Sloboda JA (2010). How does Music Evoke Emotions? Exploring the Underlying Mechanisms.. Handbook of Music and Emotion.

[27] Fornari J, Eerola T (2009). The Pursuit of Happiness in Music: Retrieving Valence with Contextual Music Descriptors..

[28] Lartillot O, Eerola T, Toiviainen P, Fornari J (2008). Multi-feature modeling of pulse clarity: Design, validation, and optimization..

[29] Fornari J, Eerola T (2008). Estimating the Perception of Complexity in Musical Harmony..

[30] Lartillot O, Eerola T, Toiviainen P, Fornari J (2008). An Integrated Framework for Onset Detection, Tempo Estimation and Pulse Clarity Prediction..

[31] Juslin PN, Karlsson J, Lindstrom E, Friberg A, Schoonderwaldt E (2006). Play it again with feeling: Computer feedback in musical communication of emotions.. Journal of Experimental Psychology-Applied.

[32] Drake C, Palmer C (2000). Skill acquisition in music performance: relations between planning and temporal control.. Cognition.

[33] Palmer C, Meyer RK (2000). Conceptual and motor learning in music performance.. Psychological Science.

[34] Bharucha JJ, Curtis M, Paroo K (2006). Varieties of musical experience.. Cognition.

[35] Droit-Volet S, Meck WH (2007). How emotions colour our perception of time.. Trends Cogn Sci.

[36] Packard MG, Wingard JC (2004). Amygdala and “emotional” modulation of the relative use of multiple memory systems.. Neurobiology of Learning and Memory.

[37] Wingard JC, Packard MG (2008). The amygdala and emotional modulation of competition between cognitive and habit memory.. Behav Brain Res.

[38] Grindea C (2001). The Phenomenon of “peak Experience” or “the flow” in Musical Performance.. The European Journal for Pianists and Piano Teachers.

[39] Eigeldinger J-J (1986). Chopin pianist and teacher; Press CU, editor..

[40] Sloboda JA (2000). Individual differences in music performance.. Trends in Cognitive Sciences.

[41] Levesque J, Eugene F, Joanette Y, Paquette V, Mensour B (2003). Neural circuitry underlying voluntary suppression of sadness.. Biological Psychiatry.

[42] Ochsner KN, Ray RD, Cooper JC, Robertson ER, Chopra S (2004). For better or for worse: neural systems supporting the cognitive down- and up-regulation of negative emotion.. Neuroimage.

[43] Ohira H, Nomura M, Ichikawa N, Isowa T, Iidaka T (2006). Association of neural and physiological responses during voluntary emotion suppression.. Neuroimage.

[44] Kompus K, Hugdahl K, Ohman A, Marklund P, Nyberg L (2009). Distinct control networks for cognition and emotion in the prefrontal cortex.. Neuroscience Letters.

[45] Ochsner KN, Gross JJ (2008). Cognitive emotion regulation: Insights from social cognitive and affective neuroscience.. Current Directions in Psychological Science.

[46] Snyder B (2000). Music and Memory.

[47] Treisman AM, Gelade G (1980). A feature-integration theory of attention.. Cogn Psychol.

[48] Wulf G, McNevin N, Shea CH (2001). The automaticity of complex motor skill learning as a function of attentional focus.. Quarterly Journal of Experimental Psychology Section a-Human Experimental Psychology.

[49] McNevin NH, Shea CH, Wulf G (2003). Increasing the distance of an external focus of attention enhances learning.. Psychological Research-Psychologische Forschung.

[50] Higuchi MKK (2007). Dificuldades no aprendizado pianística e a neuropsicologia XVII Congresso da ANPPOM 2007.

[51] Brattico E, Jacobsen T, De Baene W, Glerean E, Tervaniemi M Cognitive vs. affective listening modes and judgments of music - an ERP study.. Biol Psychol.

