# Supplementary material for: Reciprocal Modulation of Cognitive and Emotional Aspects in Pianistic Performances
Source: PLoS One. 2011 Sep 9;6(9):e24437. doi: 10.1371/journal.pone.0024437 (PMC3170321; doi:10.1371/journal.pone.0024437)
Supplement: Table S1 — Summary statistics with interactions of all Musical feature predictions of all variables studied. We have found significant p-value in average and total RMS power, articulation, event detection and pulse clarity. * We have also found significant p-value in harmonic complexity, but as the difference among the means were not significant (cognitive 1 mean 48.7; cognitive 2 mean 47.4; affective 1 48.2; affective 2 46.4), we considered these differences irrelevant. (DOC) [file pone.0024437.s001.doc]

**Supplementary File**

**Table S 1 - Summary statistics- Predictions**

| Features |  | cog 1/2 | cog 1 / afec 1 | cog 1 / afec 2 | cog 2 / afec 1 | cog 2 / afec 2 | afec 1/2 |
| --- | --- | --- | --- | --- | --- | --- | --- |
| Average RMS power | F(3;24)= 125.4 p<0.01 | p<0.01 | p<0.01 | p<0.01 | p<0.01 | p<0.01 | p<0.01 |
| Total RMS power | F(3;24)= 89,89 p<0.01 | p<0.01 | p<0.01 | p<0.01 | p<0.01 | p<0.01 | p<0.01 |
| Articulation | F(3;24)=135.32 p<0.01 | p=1.0 | p<0.01 | p<0.01 | p<0.01 | p<0.01 | p=1.0 |
| Brightness | F(3;24)=0.236 p=0.87 | p=1.0 | p=1.0 | p=1.0 | p=1.0 | p=1.0 | p=1.0 |
| **Harmonic Complexity*** | F(3;24)=19.53 p<0.01 | p=0.01 | p=1.0 | p<0.01 | p=0.27 | p=0.05 | p<0.01 |
| Event detection | F(3;24)=13.13 p<0.01 | p=1.0 | p=0.01 | p=0.01 | p=0.09 | p<0.01 | p=1.0 |
| Key clarity | F(3;24)=3.49 p=0.03 | p=0.57 | p=1.0 | p=0.13 | p=0.62 | p=1.0 | p=0.30 |
| Mode detection | F(3;24)=1.01 p=0.40 | p=1.0 | p=1.0 | p=1.0 | p=0.14 | p=0.61 | p=1.0 |
| Pulse clarity | F(3;24)=41.27 p<0.01 | p=1.0 | p<0.01 | p<0.01 | p<0.01 | p<0.01 | p=1.0 |
| Repetition | F(3;24)=2.28 p=0.1 | p=0.48 | p=1.0 | 0.48 | p=1.0 | p=1.0 | p=1.0 |
