# Supplementary material for: Reciprocal Modulation of Cognitive and Emotional Aspects in Pianistic Performances
Source: PLoS One. 2011 Sep 9;6(9):e24437. doi: 10.1371/journal.pone.0024437 (PMC3170321; doi:10.1371/journal.pone.0024437)
Supplement: Table S2 — Summary statistics with interactions of all correlations statistics of all variables studied. We have found significant p-value in intensity and pulse clarity. We also found a near significant difference in articulation. (DOC) [file pone.0024437.s002.doc]

**Supplementary File**

**Table S2 - Summary statistics - Correlations**

| Correlations |  | affect 1/2  and cog 1/2 | affect 1/2 and affect 1 / cog 1 | cog 1/2 and  affect 1 / cog 1 |
| --- | --- | --- | --- | --- |
| Intensity | F(2;16)=23.91p<0.01 | p=0.12 | p<0.01 | p=0.02 |
| Articulation | F(2;16)=4.21 p=0.03 | p=0.35 | p=0.07 | p=0.78 |
| Brightness | F(2;16)=1.03 p=0.37 | p=0.66 | p=1.0 | p=1.0 |
| Harmonic complexity | F(2;16)=2.77 p=0.09 | p=0.09 | p=1.0 | p=0.68 |
| Event detection | F(2;16)=0.52 p=0.60 | p=1.0 | p=1.0 | p=1.0 |
| Key clarity | F(2;16)=1.03 p=0.37 | p=0.51 | p=1.0 | p=1.0 |
| Mode detection | F(2;16)=0.16 p=0.85 | p=1.0 | p=1.0 | p=1.0 |
| Pulse clarity | F(2;16)=3.02 p=0.07 | p=0.38 | p=0.04 | p=1.0 |
| Repetition | F(2;16)=0.29 p=0.13 | p=1.0 | p=0.81 | p=0.08 |
